# *Plasmodium falciparum *population dynamics during the early phase of anti-malarial drug treatment in Tanzanian children with acute uncomplicated malaria

**DOI:** 10.1186/1475-2875-10-380

**Published:** 2011-12-20

**Authors:** Anja M Carlsson, Billy E Ngasala, Sabina Dahlström, Christopher Membi, Isabel M Veiga, Lars Rombo, Salim Abdulla, Zul Premji, J Pedro Gil, Anders Björkman, Andreas Mårtensson

**Affiliations:** 1 Infectious Diseases Unit, Department of Medicine Solna, Karolinska University Hospital, Karolinska Institutet, Retzius väg 10, S-171 77 Stockholm, Sweden; 2 Department of Parasitology, Muhimbili University of Health and Allied Sciences, Dar es Salaam, Tanzania; 3 Bagamoyo Research and Training Units, Ifakara Health Research and Development Centre, Bagamoyo, Tanzania; 4 Centre for Molecular and Structural Biomedicine, CBME/IBB, LA, University of Algarve, Faro, Portugal; 5 Center for Clinical Research, Sörmland County Council, Eskilstuna, Sweden; 6 Department of Biological Sciences, The Harpur College of Arts and Sciences, Binghamton University, Binghamton, NY, USA; 7 Division of Global Health, Department of Public Health Sciences, Karolinska Institutet, Stockholm, Sweden; 8 Emergency Medicine Unit, Department of Medicine, Kullbergska Hospital, Katrineholm, Sweden; 9 Drug Resistance Unit, Division of Pharmacogenetics, Department of Physiology and Pharmacology, Karolinska Institutet, Stockholm, Sweden

**Keywords:** *Plasmodium falciparum*, Malaria, PCR, Parasite population dynamics, Artemether-lumefantrine, Anti-malarial drug trials

## Abstract

**Background:**

This study aimed to explore *Plasmodium falciparum *population dynamics during the early phase of anti-malarial drug treatment with artemisinin-based combination therapy in children with clinical malaria in a high transmission area in Africa.

**Methods:**

A total of 50 children aged 1-10 years with acute uncomplicated *P. falciparum *malaria in Bagamoyo District, Tanzania, were enrolled. Participants were hospitalized and received supervised standard treatment with artemether-lumefantrine according to body weight in six doses over 3 days. Blood samples were collected 11 times, i.e. at time of diagnosis (-2 h) and 0, 2, 4, 8, 16, 24, 36, 48, 60 and 72 h after initiation of treatment. Parasite population dynamics were assessed using nested polymerase chain reaction (PCR)-genotyping of *merozoite surface protein (msp) 1 *and *2*.

**Results:**

PCR-analyses from nine sequential blood samples collected after initiation of treatment identified 20 and 21 additional genotypes in 15/50 (30%) and 14/50 (28%) children with *msp1 *and *msp2*, respectively, non-detectable in the pre-treatment samples (-2 and 0 h combined). Some 15/20 (75%) and 14/21 (67%) of these genotypes were identified within 24 h, whereas 17/20 (85%) and 19/21 (90%) within 48 h for *msp1 *and *msp2*, respectively. The genotype profile was diverse, and varied considerably over time both within and between patients, molecular markers and their respective families.

**Conclusion:**

PCR analyses from multiple blood samples collected during the early treatment phase revealed a complex picture of parasite sub-populations. This underlines the importance of interpreting PCR-outcomes with caution and suggests that the present use of PCR-adjustment from paired blood samples in anti-malarial drug trials may overestimate assessment of drug efficacy in high transmission areas in Africa.

The study is registered at http://www.clinicaltrials.gov with identifier NCT00336375.

## Background

Polymerase chain reaction (PCR) adjusted parasitological cure rate is the recommended primary end-point in clinical trials of anti-malarial drugs conducted in Africa [1]. PCR adjustment is achieved by comparing well characterized and highly polymorphic genetic markers of *Plasmodium falciparum *in a stepwise manner from one blood sample collected at time of enrolment (pre-treatment sample) with another blood sample collected at time of parasite recurrence during follow-up after treatment, i.e. paired blood samples [2]. The genotypes identified in these respective single day blood samples are distinguished from each other based on size polymorphism. Detection of the same genotype in both samples indicates treatment failure (recrudescence), whereas the absence of any genotype match suggests that the recurrent infection arose from a new inoculation/infection (treatment success)[2]. However, PCR adjusted outcome should be interpreted with caution due to inherent limitations of the technique and constraints imposed by the biology of the parasite [3].

A key issue is whether PCR analysis from paired blood samples is robust enough to ensure an accurate estimation of anti-malarial drug efficacy in clinical trials conducted in high endemic areas in Africa [4,5]. This question has been raised based on observations that children living in high-transmission areas often carry complex infections with a mixture of genetically distinct *P. falciparum *parasite sub-populations. Asymptomatic infections are characterized by extensive dynamics in detection of the individual parasite genotypes with PCR technique over time, often following a 48 h pattern [6], but sometimes appearing and disappearing within hours [7]. However, the importance and magnitude of a similar phenomenon in children with acute uncomplicated malaria in high-transmission areas remains incompletely understood. In adult travelers returning from endemic areas with clinical malaria Färnert *et al. *reported a consistent genotype pattern during consecutive day blood sampling [8], whereas Jafari *et al. *detected different genotypes at different time points using a quantitative method [9]. In Gabonese children treated with intravenous quinine the same genotype pattern was observed during the infection, but in some patients alleles disappeared and reappeared over time [10]. However, the complexity of infection in this small cohort was relatively low with 7/17 (41%) children harbouring multiclonal infections as assesses with the *msp2 *marker only.

Mårtensson *et al. *have previously reported a study assessing the influence of consecutive daily blood sampling, i.e. 4 consecutive days at enrolment and initiation of treatment and 2 consecutive days at recurrent parasitaemia, on PCR adjusted cure rates in 106 children with uncomplicated *P. falciparum *malaria in a high-transmission area in Tanzania [11]. PCR analyses identified at least one additional genotype in 25% of the patients during 3 days after enrolment and in 13% on the second day of recurrent parasitaemia, which were non-detectable at day of enrolment and initial day of recurrent parasitaemia, respectively. This may reflect parasite population dynamics of *P. falciparum *or limitations of the PCR-technique to detect all minority genotypes present due to e.g. template competition in complex infections [3,11].

Based on these previous findings the present study was undertaken to explore potential parasite population dynamics during the early phase of anti-malarial drug treatment with artemisinin-based combination therapy in African children with clinical malaria in more detail.

## Methods

### Study site, design and ethics

The study was conducted in June 2006 in Fukayosi Primary Health Care Centre, Bagamoyo District, Tanzania. The study site is located in a rural area, where *P. falciparum *is the predominant malaria species and *Anopheles gambiae *complex the main vector. At the time of the trial malaria transmission was high, i.e. with an overall slide positivity rate of approximately 40%, and perennial with peaks related to the seasonal rainfalls. The timing of the trial was chosen to coincide with the local yearly peak in malaria transmission following the main rainy season in March-May.

By the time of the trial sulphadoxine-pyrimethamine was the first- and amodiaquine second-line treatment for uncomplicated malaria in the study area. No artemisinin-containing therapies were available through the government health care services in the district during the conduct of the trial.

The primary objective of the study was to explore parasite population dynamics in children with acute uncomplicated malaria during 72 h after diagnosis and initiation of anti-malarial drug treatment. Secondary objectives included determining parasite clearance according to microscopy and PCR. The primary end-point was to compare the total number of *P. falciparum *genotypes detected pre-treatment with the accumulated number of genotypes identified per patient in all blood samples collected during the trial. The study was considered as exploratory, which precludes a sample size calculation. A sample of 50 children was predefined.

The trial was conducted in accordance with the Declaration of Helsinki and Good Clinical Practice. It was granted ethics clearance by the National Institute for Medical Research, Dar es Salaam, Tanzania, and the Regional Ethics Committee, Stockholm, Sweden. Informed consent was obtained from parents/guardians of all children enrolled. The study is registered at Clinical Trials [12], with identifier NCT00336375.

### Patients and procedures

Patients were recruited among children presenting at the study site with symptoms and signs compatible with acute uncomplicated malaria. Inclusion criteria were: a finger-prick screening blood slide with presence of *P. falciparum *asexual parasites with a density of 2,000-200,000/μL; age 1-10 years; axillary temperature ≥ 37.5°C or a history of fever in the preceding 24 h; haemoglobin (Hb) ≥ 70 g/L; informed consent; and a successful insertion of an intra-venous cannula in an ante-brachial vein. Exclusion criteria were: any cause of fever other than malaria; symptoms/signs of severe malaria; any danger signs; any serious underlying disease; intake of anti-malarial drugs within the last 14 days; known allergy to artemether-lumefantrine; and daily maximum number of five enrolments completed due to limited number of beds available in the study site.

The inclusion criteria used in this trial divert slightly from the general standards used in efficacy trials of anti-malarial drugs conducted in Africa, which usually enrol children <5 years of age with Hb ≥ 50 g/L [13]. However, the adjustments in inclusion criteria made with regards to age and Hb in this study were justified by the frequent blood sampling and the need of insertion of an intra-venous cannula.

All children excluded during the screening phase were referred to the clinician in charge of the health facility, who treated the patients according to standard practice and national guidelines.

The enrolled patients were hospitalized and randomly assigned treatment with artemether-lumefantrine (Coartem; Novartis), either accompanied with or without intake of 200 ml of full cream milk together with each drug dose. The purpose of the randomization procedure was for a pharmacokinetic and pharmacodynamic study, which has been reported elsewhere [13], where all patients participated in parallel. In the present report all enrolled patients have been analysed as a single group.

All drug doses were administered by a study nurse under supervision of the study physician at 0, 8, 24, 36, 48 and 60 h. Children weighing 5-14 kg, 15-24 kg and 25-34 kg were given one, two and three tablets per dose, respectively, each tablet containing 20 mg artemether and 120 mg lumefantrine. If a child vomited within 30 min after ingestion of the study drug, the dose was repeated.

In-patient follow-up duration was 72 h. All clinical data were recorded in a Case Record Form (CRF). Clinical assessment and blood sampling were performed in total 11 times for each participant, i.e. at the time of diagnosis (-2 h) and 0, 2, 4, 8, 16, 24, 36, 48, 60 and 72 h post-treatment. The blood sampling always included both a thick blood smear for microscopy and collection of circa 100 μL of blood on a filter paper (3 MM; Whatman) for genotyping. The screening blood sample (-2 h) was collected from a finger-prick (capillary blood). To reduce the discomfort due to the multiple blood sampling during the trial an intra-venous cannula was inserted in an ante-brachial vein just prior to enrolment and initiation of treatment (0 h), from where the remaining blood samples were to be collected (venous blood). Thus, the two pre-treatment samples (-2 and 0 h) arose from different sampling routes, capillary and venous, respectively, whereas the remaining nine blood samples collected after initiation of treatment arose from the venous route.

Giemsa-stained thick blood slides were examined directly in the field by an experienced microscopist by counting parasites against 200 white blood cells (WBC). Parasite density was quantified (parasites/μL) by assuming an average of 8,000 leucocytes per μL of blood. A negative blood slide was defined as the absence of any asexual parasite after examining 200 oil-immersion fields. Quality control was performed for all blood slides centrally (Ifakara Health Research and Development Centre, Bagamoyo Research and Training Unit) by an independent microscopists blinded to the initial blood slide results.

All blood samples for genotyping were collected on filter papers, thoroughly dried and put in individual zipper plastic bags. After completion of the field trial they were transported to Karolinska Institutet, Sweden, where the genotyping was performed.

At discharge from the study all participants received an insecticide treated net free of charge.

### Molecular analyses

DNA was extracted from all blood samples collected on filter paper using the ABI PRISM^® ^6100 Nucleic Acid PrepStation (Applied Biosystems, Fresno, CA, USA) as previously described [14]. Nested PCR genotyping was performed both for the *msp1 *and *msp2 *marker, considered to be the two most informative genetic markers for assessment of multiplicity of infection [15,16]. The respective initial amplification was followed by individual nested PCR reactions using family specific primers for *msp1 *(KI, MAD20 and R033), and *msp2 *(FC27 and IC), according to previously described standard protocols [17]. The *msp1 *and *msp2 *PCR products were loaded on 3% and 2% agarose gels, respectively, stained with ethidium-bromide, separated by electrophoresis and visualized under UV trans-illumination (GelDoc^®^, Biorad, Hercules, Ca, USA). All blood samples collected from the same patient were processed in parallel during all aspects of the laboratory work, from DNA extraction to separation of PCR products on agarose gels. At least 2 positive controls from different *P. falciparum *laboratory strains as well as 1 negative control were incorporated in each PCR run.

An adequate PCR outcome was defined for each patient, as the presence of amplified DNA in at least one genetic marker in both pre-treatment samples (-2 and 0 h), together with detectable PCR products in the positive controls. For patients not fulfilling this definition a repeat PCR run including all blood samples was performed with an increased DNA template in the initial amplification only, i.e. 3 μL instead of 1 μL in a total reaction volume of 20 μL. If an adequate PCR outcome could not be retrieved after this second PCR attempt, DNA was re-extracted and the PCR analysis repeated according to the initial protocol.

Analyses of number of genotypes and size polymorphism were digitalized using Quantity One^® ^software (Biorad, Hercules, Ca, USA). Individual genotypes were defined by binning 20 base-pairs intervals together. Initially, the median genotype for each family of the respective genetic markers was identified. The absolute size of the identified median band +/- 10 base-pairs formed the initial bin. Thereafter, each 20 base pair interval below and above the median band were defined as representing a distinct genotype.

### Statistical analyses

Data were entered, validated and analysed using SPSS 15.0 software. Arithmetical means were calculated for all continuous baseline demographic variables, except for asexual parasite density (geometric mean). Continuous variables were compared between the two pre-treatment samples as well as between pre-treatment (-2 and 0 h combined) and samples collected after treatment initiation using paired sample *t*-test. Proportions of binary outcomes for related samples were compared using McNemar's *χ*^2 ^test. Statistical significance was defined as a *P*-value ≤0.05. Where applicable, 95% confidence intervals (CI) were calculated. The primary end-point was analysed for all patients enrolled in the study using the intention to treat approach. To adjust for the differences in methodology for pre-treatment blood sampling, capillary versus venous, the genotyping outcome for these two blood samples (-2 and 0 h) were combined in the analyses of the primary end-point of the study.

## Results

### Screening, baseline/pre-treatment characteristics and compliance

Out of 277 children screened for eligibility, 148 (53%) had a positive blood-slide for *P. falciparum*, but 98 (66%) had to be excluded for the following reasons: parasite density >200,000/μL (N = 7); parasite density <2,000/μL (N = 25); age <1 year (N = 5); age >10 years (N = 1); Hb <70 g/L (N = 11); severe malaria (N = 2); severe malnutrition (N = 1); refused consent (N = 13); recent intake of anti-malarial drugs (N = 1); not possible to establish an intra-venous line (N = 11); and maximum daily number of admissions completed (N = 21).

A total of 50 children were thus enrolled, including 31 (62%) girls and 19 (38%) boys, of whom 33 (66%) were below 5 years of age. Mean age and weight was 51 (range 12-119) months and 14.3 (range 8.0-30.0) kg, respectively. Some 37/50 (74%) children were febrile with a temperature of ≥37.5°C at screening (-2 h). At this time point 27/50 (54%) children received antipyretic treatment (paracetamol). A statistically significant decrease in mean temperature was observed between the two pre-treatment measurements (-2 and 0 h), from 38.5°C to 37.9°C (*p *= 0.004; paired sample *t*-test). In contrast, parasite geometric mean showed a statistically significant increase from 21,705 (range 2,120-192,000) parasites/μL to 37,710 (range 2,120-200,400) parasites/μL (*p *= 0.038; paired sample *t*-test) between -2 and 0 h. A majority of the patients, 28/50 (56%), had an at least two-fold difference in parasite density between -2 and 0 h, of whom 21 showed an increase of more than 100% and 7 a decrease of at least 50%.

No death, development of severe malaria or any other serious adverse event occurred during the trial. Forty-nine children completed the stipulated 72-h follow-up. They all had adequate clinical and parasitological response by 72 h. The remaining child completed follow-up until 48 h, but was then withdrawn due to guardians' request. This child had a negative blood slide at discharge and had received five of the total six artemether-lumefantrine doses under supervision during study participation. The final drug dose was provided to the parents to be administered at home.

### Parasite positivity rates, PCR success rate and parasite clearance

From the 50 children enrolled a total of 548 blood samples were collected for microscopy and genotyping, of which 312 (57%) had a positive blood slide for asexual *P. falciparum *and 365 (67%) a positive PCR amplification in at least one genetic marker (McNemar's *χ*^2 ^test 32.6, *p *< 0.001). The respective *msp1 *and *msp2 *markers were positive in 330 (60%) and 325 (59%) of the 548 samples. The corresponding PCR positivity rates for the individual families of *msp1 *were for KI: 213 (39%) MAD20: 114 (26%) and R033: 152 (28%), and for *msp2 *FC27: 206 (38%) and IC: 265 (48%), respectively. A positive PCR result for at least one genetic marker was also retrieved in 68/236 (29%) blood samples, when microscopy was reported negative.

The overall PCR success rate, defined as the ability to amplify parasite DNA in either *msp1 *or *msp2 *when microscopy showed asexual *P. falciparum *parasitaemia, was 297/312 (95%), and for the individual markers 281/312 (90%) and 279/312 (89%) for *msp1 *and *msp2*, respectively. An adequate PCR outcome could be established after the first PCR attempt in 46/50 (92%) patients. Four patients (ID 5, 8, 11 and 20) required one repeat PCR run, whereafter they fulfilled the definition of adequate PCR outcome. The 15 samples with overall PCR failure were distributed among eight patients, of whom four (ID: 5, 8, 11 and 26) had 2-4 negative samples and the remaining 4 (Patient ID: 15, 34, 39 and 45) had one negative sample each.

**Figure 1:**
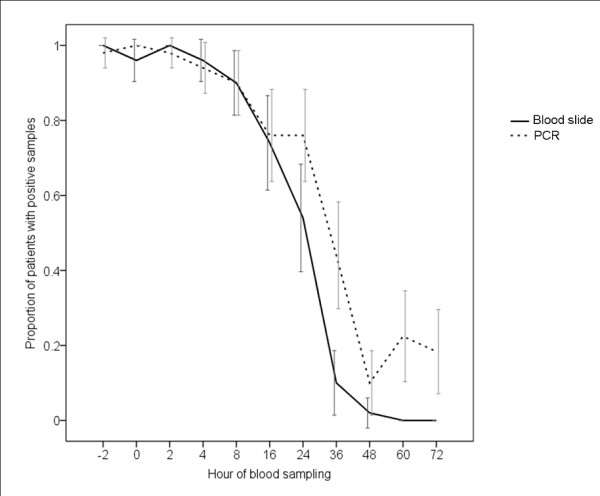
***P. falciparum *clearance measured as proportion of patients with positive blood slides (asexual parasitaemia) and PCR results (defined as a positive amplification in at least one genetic marker, i.e. *msp1 *or *msp2*) at each blood sampling point during the study**. (Error bars represent 95% confidence intervals)

Parasite clearance (Figure 1) measured as proportion of patients with positive microscopy was rapid and effective following artemether-lumefantrine treatment. By 24 h after initiation of treatment 27/50 (54% [95% CI 40-68%]) patients had a positive blood slide, whereas 41/50 (82% [95% CI 71-93%]) remained positive by PCR. From this time point and onwards PCR clearance was generally longer as compared with microscopy clearance, leaving 11/50 (22%) and 9/50 (18%) children with a positive PCR result at 60 and 72 h, respectively, when no samples remained positive by microscopy.

### Genotype diversity and distribution

**Figure 2:**
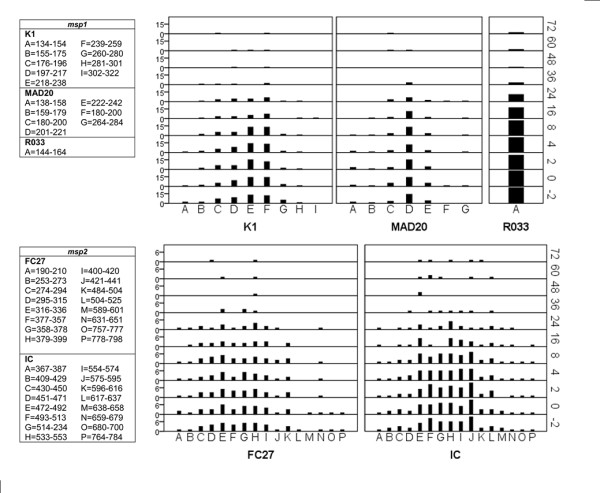
**Overall distribution and frequencies of individual genotypes during follow-up in the entire cohort**. Individual genotypes are represented on the x-axis with the frequency at which they occur (left y-axis) by each blood sampling point during the study (right y-axis)

Total numbers of detectable genotypes and overall basepair ranges as well as relative prevalences of the most detected genotype for the respective families within *msp1 *and *msp2 *are presented in Table 1. Overall distribution and frequencies of individual genotypes during follow-up is presented in Figure 2.

**Table 1:** Basepair range and number of detected genotypes of the respective families of *msp1 *and msp2

	*msp1*			*msp2*	
	
	K1	MAD20	R033	FC27	IC
**Basepair range**	141-318	148-265	147-160	198-783	368-768

**Basepair interval & prevalence of the most detected genotype among positive PCR samples**	239-259 82/284 (29%)	201-221 89/159 (56%)	144-164 152/152 (100%)	316-336 35/226 (15%)	575-595 48/345 (14%)

**Number of genotypes**	9	7	1	16	16

**Total number of genotypes**	= 17			= 32	

### Complexity of infection and parasite population dynamics

The complexity of infection pre-treatment (-2 and 0 h combined) was high, with 32/50 (64%) and 37/50 (74%) patients carrying multiple genotypes in *msp1 *[median 2, range 0-7] and *msp2 *[median 2, range 0-7], respectively. A total of 16/50 (32%) and 12/50 (24%) patients had different genotype patterns detected in the two pre-treatment samples for *msp1 *and *msp2*, respectively. This comprised 19 genotypes in *msp1 *and 18 in *msp2*. A total of eight *msp1 *genotypes (6 K1, 1 MAD20 and 1 R033) and eight *msp2 *(3 FC27 and 5 IC) were detected -2 h but absent 0 h, whereas 11 (4 K1, 6 MAD20 and 1 R033) and 10 (3 FC27 and 7 IC) genotypes, respectively, appeared at 0 h without having been identified -2 h.

In the nine sequential blood samples collected after initiation of treatment, *msp1 *genotyping identified a total of 20 (10 KI, 7 MAD20 and 3 R033) genotypes in 15/50 (30%) patients, which were non-detectable pre-treatment (-2 and 0 h combined). Of these 15 patients, 11 had one, 3 had two and 1 had three new genotypes, respectively. Similarly, *msp2 *genotyping after initiation of treatment identified 21 (6 FC27 and 15 IC) new genotypes in 14/50 (28%) children, of whom seven children each had one and two new genotypes, as compared with the combined pre-treatment outcome. Including these 20 and 21 new genotypes as part of the overall complexity of infection the proportion of children harbouring multiple genotypes increased from 32/50 (64%) and 37/50 (74%) to 36/50 (72%) and 39/50 (78%) for *msp1 *and *msp2*, respectively, without affecting the median number of genotypes detected in either of the genetic markers.

**Figure 3:**
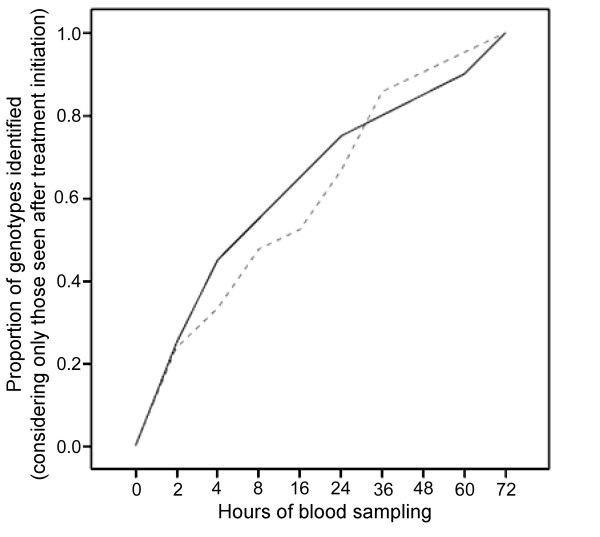
Accumulated proportion of primary identification during follow-up of *msp1 *(N = 20, solid line) and *msp2 *(N = 21, broken line) genotypes exclusively identified after initiation of treatment

Figure 3 shows the accumulated proportion of the initial detection over time of the *msp1 *and *msp2 *genotypes exclusively identified after initiation of treatment. Of these 20 and 21 genotypes, 15 (75%) and 14 (67%) were identified within 24 h, whereas 17 (85%) and 19 (90%) were detected within 48 h for *msp1 *and *msp2*, respectively.

**Figure 4:**
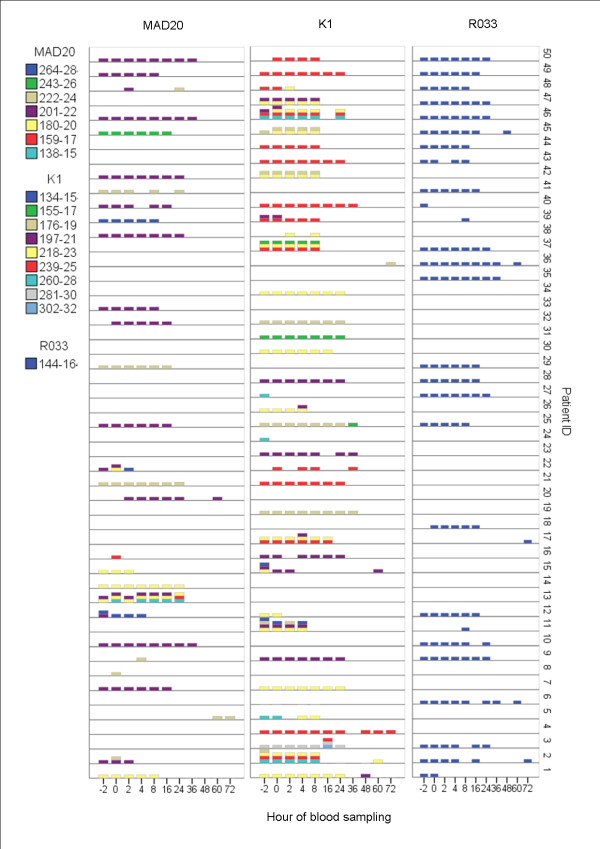
Distribution of detectable *msp1*genotypes over time for each study participant

PCR outcome for each participant during the entire study are presented in Figures 4 (*msp1*) and 5 (*msp2*). The genotype profile fluctuated considerably over time, both within and between patients, molecular markers and their respective families making it difficult to categorize or quantify specific genotype patterns. However, some general genotype patterns were identified:

**Figure 5:**
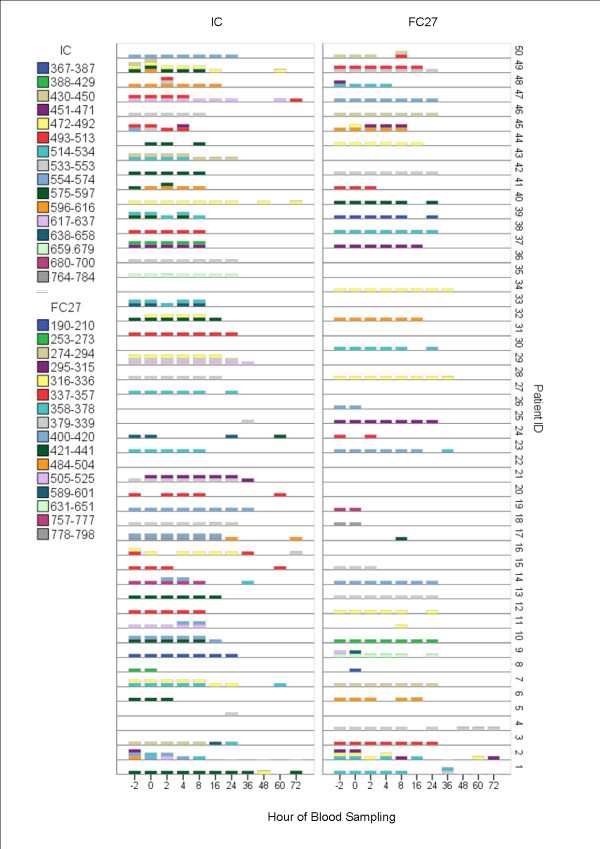
Distribution of detectable *msp2*genotypes over time for each study participant

a) Continous and fading; one or several genotypes present pre-treatment, persist/s for some time and finally fade/disappear.

b) Continuous and fading with appearance of random genotypes; this pattern is similar to pattern a (above), but with additional random band/s appearing at any time-point during follow-up.

c) Intermittent; one or several genotypes present pre-treatment disappear and reappear over time.

d) Random; no clear pattern can be discerned.

**Figure 6:**
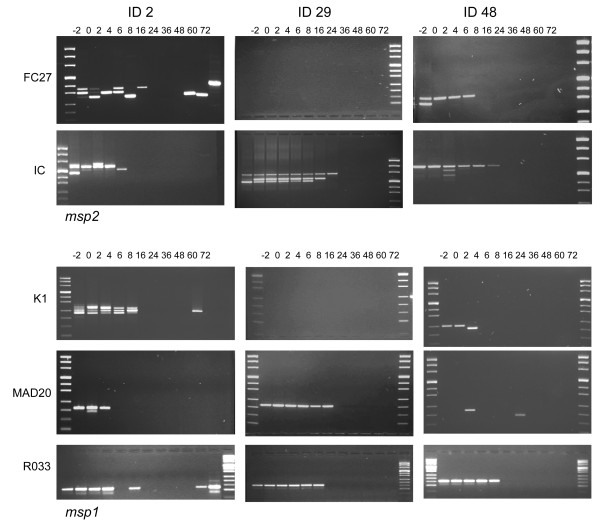
**Example of different PCR patterns**. Patient ID 2 is an example of intermittent genotype pattern, whereas ID 29 shows continuous fading pattern in all markers and ID 48 a continuous fading pattern with random bands in *msp2 *IC and a random pattern in *msp1 *MAD20. *Patient ID 2 is followed by a positive control for *msp2 *(FC27) and *msp1 *(R033)

Example of genotype patterns a, b and c (above) is presented in Figure 6. A majority of patients appeared to have continues and fading genotype pattern (a). However, genotyping results from one patient often revealed a combination of the above patterns. Patients carrying complex infections were also likely to have one or few genotypes detected more frequently than others throughout the infection, suggesting the presence of dominant genotype/s. A minority of patients showed extensive genotype dynamics, e.g. patient ID 2 (Figure 6). This 72 months old child had a low grade asexual parasitaemia of 2120/μL at enrolment, and never a temperature exceeding 37.5°C during follow-up.

## Discussion

The introduction of PCR genotyping in clinical drug trials of anti-malarial drugs has significantly improved the distinction between treatment failure (recrudescence) and reinfections among recurrent infections post-treatment. The PCR technique is in theory simple and straight-forward. However, in real life situations the technique is well known to have limitations, particularly to amplify minority clones in complex infections, which is a common feature in children residing in high-transmission areas in Africa, but also due to constraints imposed by the biology of the parasite, with sequestration in the deep vascular system during the later part of the erythrocytic life cycle [3]. However, the accuracy of PCR-adjusted results in anti-malarial drug trials do not only rely on the PCR technique *per se*, but on a chain of different events preceding the DNA amplification and separation of PCR products, from blood sampling in the field and duration of storage to extraction of parasite DNA, each being associated with potential risk of influencing the final PCR outcome and thus the interpretation of the efficacy of the drugs at test [15,18]. Therefore, PCR results should be interpreted with caution [3].

In this study, parasite population dynamics, as assessed with PCR genotyping of *msp1 *and *msp2 *during the early phase of anti-malarial drug treatment in children with acute uncomplicated *P. falciparum *infections in a high-transmission area in Tanzania, were explored in detail. The results indicate that a single blood sample may not provide a complete picture of all parasite subpopulations present in children with acute uncomplicated malaria in high-transmission areas in Africa. This is in agreement with our previous observations [11]. However, PCR analyses from multiple blood samples collected during the early phase of anti-malarial drug treatment in this trial revealed a more complex picture of parasite sub-populations, as compared with the daily consecutive blood samples reported by Mårtenson *et al. *[11].

Similarly with Mårtensson *et al. *previous report [11], a majority of the additional genotypes exclusively detected after initiation of treatment were identified within 24 h after enrolment, at a time when 41/50 (82%) children still had a positive PCR amplification in at least one genetic marker. However, the most pronounced difference in detected genotype pattern was observed between the two pre-treatment samples (-2 and 0 h). Similarly, a majority of patients had an at least a twofold difference in parasite density before initiation of treatment. These observations may reflect a dynamic/chaotic situation in untreated children with acute malaria infections, where individual parasite genotypes increase and decrease in a non-synchronized way in the peripheral blood and thus within short time periods may be present in different proportions of the overall parasitaemia. However, considering that the blood collection from these two sampling points arose from different routes (capillary versus venous), the observations may also be due to differences in methodology. Interestingly, no data are, to the authors knowledge, available on comparison of genotyping results from capillary and venous blood [2].

The child (ID 2) with the most complex genotype pattern in our trial, presented both with a low grade parasitaemia and fever. Despite fulfilling the study inclusion criteria, this child may have suffered from a self-limiting cause of fever other than malaria. If so, the genotype pattern observed may represent an asymptomatic infection rather than parasite population dynamics in acute uncomplicated malaria.

It should be underlined that parasite population dynamics were assessed based on size polymorphisms of PCR fragments after separation with electrophoresis on agarose gels. This method still remains one of the recommended methods in anti-malarial trials to distinguish treatment failures (recrudescence) from new infections [2] and is still widely used in clinical trials. However, its discriminatory power is generally considered to be inferior to more recently developed methods, e.g. capillary electrophoresis [2,19]. This may have influenced the estimation of parasite population dynamics in this study. Liljander *et al. *found that individual measurements of the same PCR fragments run in different lanes on the same agarose gel varied from 2 to 16 basepairs [18]. To account for this a conservative approach was applied when defining individual genotypes by binning 20 basepairs together.

## Conclusions

The results from this study indicate that PCR analyses from multiple blood samples collected during the early treatment phase revealed a complex picture of parasite sub-populations. However, it remains open whether the complex genotype pattern observed in our study reflects true parasite population dynamics or limitations of the PCR-technique to detect all minority genotypes present due to e.g. template competition in complex infections. The results underline the importance of interpreting PCR outcomes with caution and suggest that the present use of PCR-adjustment from paired single day blood samples in anti-malarial drug trials may overestimate assessment of drug efficacy in high transmission areas in Africa.

## Competing interests

We declare that we have no conflict of interest.

## Authors' contributions

AMC performed PCR-genotyping, data analyses and interpretation and assisted in writing of the report. BEN, SD and CM, planned and conducted the field trial. LR participated in the interpretation of data and in writing of the report. SA and ZP participated in study planning, coordination the field trial and assisted in writing of the report. JPG, MIV and AB contributed to study design, planning and coordination, interpretation of results, and preparation of the report. AM designed and planned the study, conducted the field trial, performed data analyses and interpreted the results, and wrote the report. All authors read and approved the final manuscript.
